# An Echocardiography Training Program for Improving the Left Ventricular Function Interpretation in Emergency Department; a Brief Report 

**Published:** 2017-06-15

**Authors:** Mary Jacob, Hamid Shokoohi, Fabith Moideen, Amelia Pousson, Keith Boniface

**Affiliations:** 1 Department of Emergency Medicine, George Washington University, Washington DC. USA.; 2 Department of Emergency Medicine, Baby Memorial Hospital, Calicut, Kerala, India. shokoohi@gwu.edu (corresponding author)

**Keywords:** Echocardiography, ultrasonography, ventricular Function, Left, educational techniques

## Abstract

**Introduction:**

Focused training in transthoracic echocardiography enables emergency physicians (EPs) to accurately estimate the left ventricular function. This study aimed to evaluate the efficacy of a brief training program utilizing standardized echocardiography video clips in this regard.

**Methods:**

A before and after design was used to determine the efficacy of a 1 hour echocardiography training program using PowerPoint presentation and standardized echocardiography video clips illustrating normal and abnormal left ventricular ejection fraction (LVEF) as well as video clips emphasizing the measurement of mitral valve E-point septal separation (EPSS). Pre- and post-test evaluation used unique video clips and asked trainees to estimate LVEF and EPSS based on the viewed video clips.

**Results:**

21 EPs with no prior experience with the echocardiographic technical methods completed this study. The EPs had very limited prior echocardiographic training. The mean score on the categorization of LVEF estimation improved from 4.9 (95% CI: 4.1-5.6) to 7.6 (95%CI: 7-8.3) out of a possible 10 score (p<0.0001). Categorization of EPSS improved from 4.1 (95% CI: 3.1-5.1) to 8.1 (95% CI: 7.6- 8.7) after education (p<0.0001).

**Conclusions:**

The results of this study demonstrate a statistically significant improvement of EPs’ ability to categorize left ventricular function as normal or depressed, after a short lecture utilizing a commercially available DVD of standardized echocardiography clips.

## Introduction

Assessment of left ventricular function by point-of-care echocardiography is of particular importance in differentiating the causes of some presentation such as hypotension and dyspnea in emergency department. Research has demonstrated that focused training in transthoracic echocardiography enables EPs to accurately classify left ventricular ejection fraction (LVEF) as normal, depressed, or severely depressed (1, 2). 

Over 20 years ago, Plummer et al published their sentinel paper on the impact of point-of-care echocardiography in the evaluation of penetrating cardiac trauma (3). Since that time, focused clinician-performed echocardiography has been found to be useful in the assessment of patients with cardiac arrest, suspected massive pulmonary embolism, and hypotension (4, 5).

There are several technique for echocardiographic evaluation of LVEF, including Simpson’s rule, wall motion index, and subjective visual estimation. McGowan et al. performing a systematic review found that none of the three mentioned methods had superiority to others in estimation of LVEF (6). Simpson’s method requires significant experience, and can often be limited by technically suboptimal examinations with indistinct endocardial borders. However, visual estimation of LVEF is a commonly employed technique and correlates well with ventriculography (7). 

Mitral valve E-point septal separation (EPSS), is another easy-to-obtain echocardiographic parameter that correlates inversely with LVEF. EPSS is measured as the minimal distance between the anterior mitral valve leaflet and the interventricular septum in the parasternal long view during diastole using M-Mode on echocardiography. Secko et al. showed that junior EPs could obtain EPSS measurements that correlated with visual estimates of LVEF (8).

We created a training program in point-of-care echocardiography utilizing echocardiography video clips from patients with known LVEF. In this method the trainees were taught to focus on the anterior mitral valve’s motion relative to the septum. The aim of this study was to evaluate the efficacy of this brief educational training program in improving LVEF and EPSS interpretation by EPs.

## Methods

***Study design and setting***

A before and after design was used to determine the efficacy of a brief educational echocardiography training program on interpretation of left ventricular function with regards to LVEF and EPSS. The study was conducted in two urban adult academic Emergency Departments in Baby Memorial and Malabar Institute of Medical Sciences (MIMS) Hospitals in Calicut, Kerala, India. Data were collected during the academic year of 2014-2015. The George Washington (GW) University Institutional Review Board approved the study, and letters of support were obtained from the two participating hospitals in India. Both hospitals gave written permission for the study and reviewed the institutional review board forms prior to study commencement. The study investigators were two attending EPs and one senior resident from an ACGME approved Emergency Medicine residency training program. No patients were involved and informed consent was obtained from the EPs involved in the study. 

***Participants***

The study population was the emergency physicians in post graduate training at the mentioned Hospitals. All emergency physicians in-training were included in the study and medical students and faculty in practice were excluded. 

***Outcomes***

The primary goal of this study was to determine the efficacy of a brief educational training program utilizing standardized echocardiography video clips in improving EPs’ skills in categorizing LVEF as normal (>50%), depressed (30-50%), or severely depressed (<30%) based upon a single parasternal long axis view. The secondary goal was to classify EPSS as normal (< 8 mm) or increased (≥ 8 mm) based on B-mode images. 

**Table 1:** Demographic characteristic of participants

**Characteristics**	**No (%)**
**Year of Training**	
1	8 (38.09)
2	7 (33.33)
3	3 (14.28)
Not reported	3 (14.28)
**Prior ultrasound training**	
Yes	10 (47.61)
No	8 (38.09)
Not reported	3 (14.28)
**Ultrasound use in last month**	
Non-user	5 (23.80)
User	13 (61.90)
Not reported	3 (14.28)
**Comfort level**	
Not at all	5 (23.80)
Somewhat	12 (57.14)
Very	2 (9.52)
Not reported	2 (9.52)

**Figure 1:**
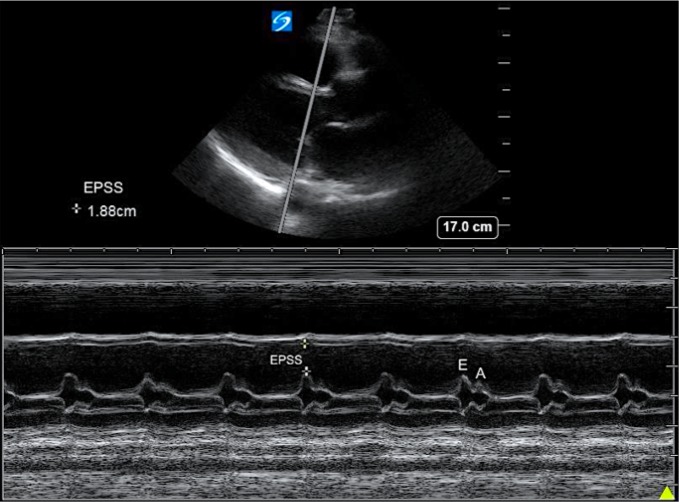
An M-mode ultrasound scan through the distal- Most aspect of the anterior leaflet of the mitral valve to trace the movement of the anterior MV leaflet through the cardiac cycle. The E and A points of each diastole are visible as asymmetric humps during each diastole.

**Figure 2:**
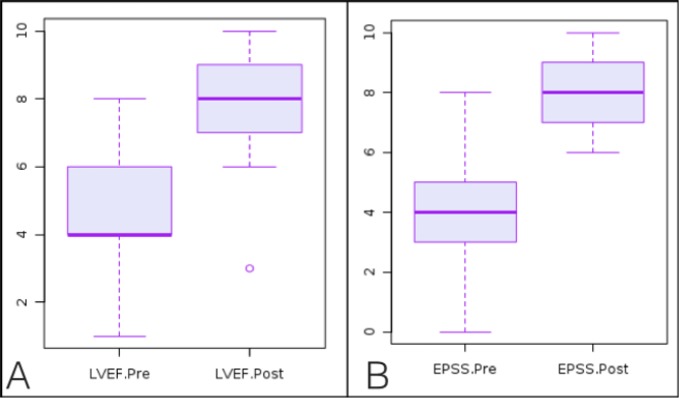
LVEF and EPSS interpretation before and after video-based module training (p < 0.0001 for both measurements). LVEF=left ventricular ejection fraction; EPSS=end point septal separation.

***Intervention***

 The EPs’ training intervention for estimating LVEF and EPSS consisted of a 1 hour didactic by the senior level emergency medicine resident who had previously completed a two-week ultrasound rotation and had undergone dedicated 2 hour echocardiography training with experienced emergency ultrasonographers. The course utilized materials consisting of a PowerPoint presentation and a number of standardized video clips illustrating normal and abnormal LVEF as well as video clips emphasizing the measurement of EPSS. The echocardiography video clips were part of a commercially available DVD with patients who had undergone contemporaneous radionuclide ventriculography, giving the LVEF. By utilizing a series of standardized examples of a wide range of ejection fractions, the training period was able to be compressed. The EPSS from the video clips was estimated from the B-mode images using calipers by the GW ultrasound quality assessment team led by an ultrasound fellowship trained attending. EPSS measurements of > 8 mm indicate poor left ventricular function (figure1).

The EPs completed a 10 question pre-test and post-test using standardized video clips of parasternal long axis view to estimate LVEF and EPSS based on these video clips from patients with known ejection fraction. Each correct answer earned 1 point and inappropriate ones zero point. 

The pre- and post-test clips, as well as the video clips in the PowerPoint presentation, were all different from one another but had the same number of normal, depressed, and severely depressed LVEF videos clips. The pre- and post- tests also reported a survey of broad demographics (year of training, prior ultrasound training, self-reported confidence in echocardiographic interpretation, prior echocardiograms training, and using ultrasound in last month). No personal identifiers were collected in this study.

***Statistical analysis***

Sample size was determined by the class size of international EPs. Data were analyzed Using Stata version 10.1 and presented as number (%) or mean ± standard deviation. A paired samples t-test was used to compare two related means. A p-value of less than 0.05 considered significant. 

## Results

32 EPs were enrolled in the post graduate EM training programs, among them 21 EPs (65.62%) were available and participated in both pre- and post-tests. The baseline characteristics of EPs and their previous experiences with ultrasound is summarized in Table 1. The EPs had very limited prior echocardiographic training aside from the cardiac view of the Focused Assessment with Sonography in Trauma (FAST) examination. 

 The mean score on the categorization of LVEF estimation improved from 4.9 (95% CI: 4.1-5.6) to 7.6 (95%CI: 7-8.3) out of a possible 10 score (p< 0.0001). Categorization of EPSS improved from 4.1 (95% CI: 3.1-5.1) to 8.1 (95% CI: 7.6- 8.7) after education (p<0.0001) (Figure 2).

## Discussion

The results of this study demonstrate a statistically significant improvement of EPs’ ability to categorize left ventricular function as normal or depressed, after a short lecture utilizing a commercially available DVD of standardized echocardiography clips. 

LVEF and EPSS are important data points in critical care and emergency medicine decision-making. Visual estimation skills in this regard typically are gained via many hours of experience at the bedside. 

In this study, a senior emergency medicine resident with skills in focused echocardiography successfully taught a group of EPs visual estimation of LVEF and EPSS using a brief training program comprised of commercially available, standardized echocardiography clips, with an emphasis on the motion of the anterior leaflet of the mitral valve as a teaching tool. 

The ability of EPs to accurately categorize LVEF has been demonstrated in the past, utilizing an extended period of time with hands-on scanning, both in the echocardiography lab as well as in the ED. Moore et al evaluated the ability of EPs with focused training in echocardiography to determine left ventricular function of hypotensive emergency department patients compared to a blinded cardiologist reviewing the echocardiogram acquired by the EP (1). Training was accomplished with a combination of didactics (six hours of videotaped instruction), time in the echocardiography laboratory under supervision of a cardiologist (greater than ten hours), and completion of complete echocardiogram study reviewed for adequacy by a cardiologist prior to beginning patient enrollment. Weighted kappa for the categorization of LVEF by EPs and cardiology in this study was 0.61 (95% CI 0.39-0.83) and the length of time required for completion of the echocardiogram was 17.5 +/- 10.8 minutes (range 4-45 minutes). The authors state in their discussion, however, that the majority of the time was spent taking M-mode measurements, border tracing, and capturing adequate views for printing, and they estimate that they could complete a five-view echocardiogram in less than five minutes focusing on LVEF. 

Randazzo et al. studied visual estimation of LVEF in an ED population as well (2). The investigators undertook additional training in limited echocardiography (three hours of didactics, review of normal and abnormal echocardiograms, and 5 proctored exams on patients who were not enrolled in the study) and compared EP LVEF category with that of a complete echocardiogram performed by cardiology. Weighted kappa for the categorization of LVEF by EPs and cardiology was 0.71 (95% CI 0.53-0.89). Subgroup analysis revealed the highest agreement (92.3%) between EPs and formal echocardiograms within the normal LVEF category, followed by 70.4% agreement in the poor LVEF category and only 47.8% in the moderate LVEF category.

This study is the first to demonstrate that a didactic session utilizing standardized echocardiography clips from patients with known LVEF can lead to improved accuracy in the determination of systolic function. Improving EP echocardiographic interpretation skills can be beneficial by rapidly identifying those patients with abnormal systolic function, utilizing the motion of the anterior leaflet of the mitral valve as a tool to more accurately assess LVEF. This study also evaluated the use of a single view echocardiogram as a physical exam adjunct in distinguishing cardiogenic from non-cardiogenic dyspnea based on ejection fraction (EF) by using a depressed EF as a marker for congestive heart failure as well as EPSS as a tool for left ventricular function delineation. 

***Limitations***

There were a number of limitations in this study. This study took place in its entirety in the classroom setting, and as such the EPs did not acquire the images themselves. Image interpretation is a separate skill set than image acquisition, and both facets of point-of-care ultrasound are critical to arriving at the correct diagnosis. In addition, EPSS is traditionally a measurement made from M-mode. This training session emphasized the concepts of anterior mitral valve leaflet motion relative to the septum that is at the core of the M-mode EPSS measurement, and these concepts can be applied in real time during B-mode imaging as a component of visual estimation. Also, the use of a solitary view of the heart can be deceiving when estimating LVEF in the presence of regional wall motion abnormalities. The video clips used in this study were older images from a commercially available digital resource. However, this did not appear to limit the EPs’ interpretation of images. The study did not seek to differentiate the incremental increase in diagnostic accuracy of LVEF determination with EPSS compared to without teaching EPSS, as the sample size was too small to allow two comparison groups. Finally, this study did not test long-term retention, which would be the next step in the evaluation of video-based training program as an adjunct to international EPs’ ultrasound training.

## Conclusions

The results of this study demonstrate a statistically significant improvement of EPs’ ability to categorize left ventricular function as normal or depressed, after a short lecture utilizing a commercially available DVD of standardized echocardiography clips. 
